# Extracellular volume imaging by T1 mapping cardiovascular magnetic resonance in patients with clinically suspected myocarditis

**DOI:** 10.1186/1532-429X-15-S1-P116

**Published:** 2013-01-30

**Authors:** Ulf K Radunski, Gunnar Lund, Mandana D Nariman, Bernhard Schnackenburg, Christian Stehning, Gerhard Adam, Stefan Blankenberg, Kai Muellerleile

**Affiliations:** 1 University Heart Center Hamburg, Hamburg, Germany; 2 Diagnostic and Interventional Radiology, University Medical Center Hamburg-Eppendorf, Hamburg, Germany; 3 Philips Research Hamburg, Hamburg, Germany; 4 Philips Healthcare Hamburg, Hamburg, Germany

## Background

Assessing the extracellular volume fraction (ECV) by T1 mapping is a promising cardiovascular magnetic resonance (CMR) approach to quantify myocardial injury. This study evaluated ECV imaging by T1 mapping CMR in patients with clinically suspected myocarditis.

## Methods

**Figure 1:**
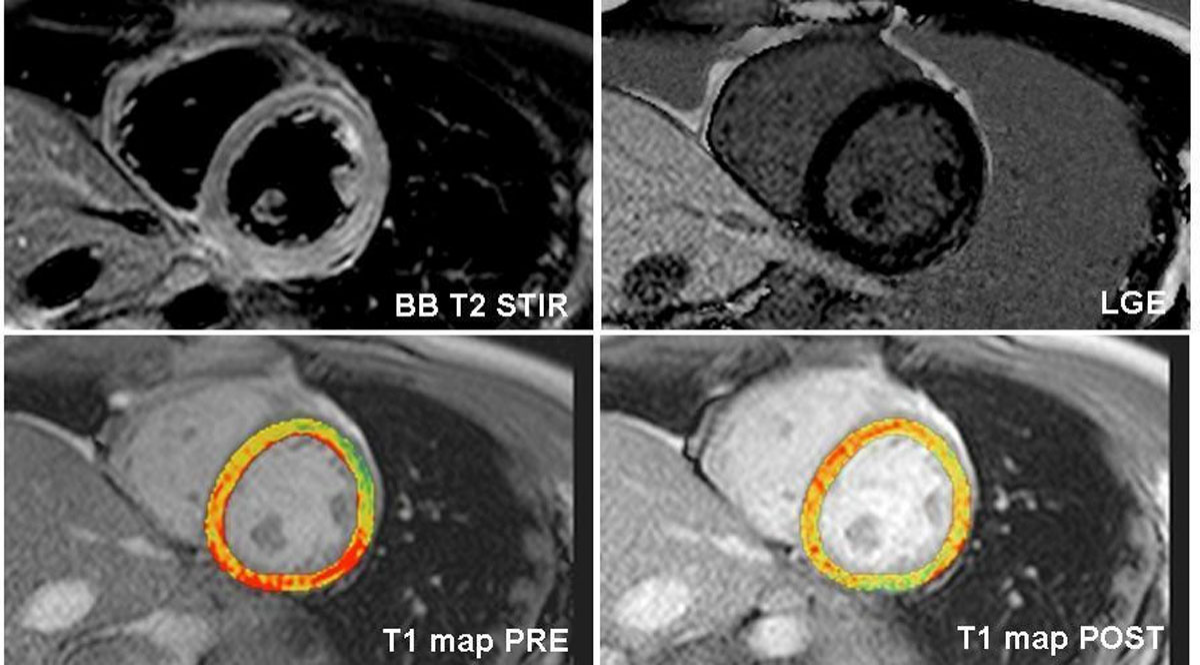


T1 mapping was implemented into a standard CMR protocol in 113 patients with clinically suspected myocarditis at 1.5 Tesla. T1 quantification was performed for ECV calculation using the modified Look-Locker inversion-recovery (MOLLI) sequence on three short-axes before and 15 minutes after administration of 0.075 mmol/kg gadolinium BOPTA, respectively. Global left ventricular ECV was calculated from T1 maps generated with a dedicated plug-in written for the OsiriX software: relaxation rates (1/T1 = R1) were calculated for myocardium and blood pool. The difference in R1 between pre- and post contrast media was calculated as ΔR1. Myocardial ECV was then estimated using the formula: ECV = 1-hematocrit * (ΔR1_myocardium_/ΔR1_blood pool_). Figure 1 demonstrates an example for T1 maps pre- and post contrast media in comparison with standard black-blood T2 STIR and late gadolinium enhancement (LGE) images.

## Results

The final study population consisted of 96 patients after exclusion of 17 patients with concomitant coronary artery disease or severe left ventricular hypertrophy. Current clinical and CMR criteria identified 64 (67 %) patients with myocarditis and 32 (33 %) patients without myocarditis in the final study population. Global ECV was significantly larger in patients with myocarditis compared to patients without myocarditis (35±6 vs. 30±5 %, p<0.0001). Furthermore, global ECV was significantly larger in patients with focal scar compared to patients without focal scar on LGE CMR (35±5 vs. 33±7 %, p<0.05). ROC analysis revealed a sensitivity of 73 % and a specificity 67 % to identify patients with myocarditis using a global ECV ≧ 32 % cut-off (AUC = 0.76, p<0.0001).

## Conclusions

Patients with myocarditis are characterized by an increased global ECV. Assessing global ECV by T1 mapping has great potential to improve the diagnostic and prognostic impact of CMR in patients with clinically suspected myocarditis.

## Funding

Orlovic Foundation

